# The influence of early selenium supplementation on trauma patients: A propensity-matched analysis

**DOI:** 10.3389/fnut.2022.1062667

**Published:** 2022-12-08

**Authors:** Yu-Cheng Chiu, Chia-Ming Liang, Chi-Hsiang Chung, Zhi-Jie Hong, Wu-Chien Chien, Sheng-Der Hsu

**Affiliations:** ^1^Division of General Surgery, Tri-Service General Hospital, National Defense Medical Center, Taipei, Taiwan; ^2^Division of Trauma Surgery and Critical Care Medicine, Tri-Service General Hospital, National Defense Medical Center, Taipei, Taiwan; ^3^Department of Medical Research, Tri-Service General Hospital, National Defense Medical Center, Taipei, Taiwan; ^4^Division of Trauma Surgery, Tri-Service General Hospital, National Defense Medical Center, Taipei, Taiwan; ^5^School of Public Health, National Defense Medical Center, Taipei, Taiwan

**Keywords:** selenium supplement, selenoproteins, trauma, pulmonary complication, length of stay

## Abstract

**Introduction:**

Oxidative stress is involved in numerous inflammatory diseases, including trauma. Micronutrients, such as selenium (Se), which contribute to antioxidant defense, exhibit low plasma levels during critical illness. This study aimed to investigate the impact of early Se supplementation on trauma patients.

**Materials and methods:**

A total of 6,891 trauma patients were registered at a single medical center from January 2018 to December 2021. Twenty trauma patients with Se supplemented according to the protocol were included in the study group. Subsequently, 1:5 propensity score matching (PSM) analysis was introduced. These patients received 100 mcg three times a day for 5 days. The primary outcome was overall survival (OS); the secondary outcomes were hospital/intensive care unit (ICU) length of stay (LOS), serologic change, ventilator dependence days, and ventilation profile.

**Results:**

The hospital LOS (20.0 ± 10.0 vs. 37.4 ± 42.0 days, *p* = 0.026) and ICU LOS (6.8 ± 3.6 vs. 13.1 ± 12.6 days, *p* < 0.006) were significantly shorter in the study group. In terms of serology, improvement in neutrophil, liver function, and C-reactive protein (CRP) level change percentile indicated better outcomes in the study group as well as a better OS rate (100 vs. 83.7%, *p* = 0.042). Longer ventilator dependence was found to be an independent risk factor for mortality and pulmonary complications in 6,891 trauma patients [odds ratio (OR) = 1.262, 95% confidence interval (CI) = 1.039–1.532, *p* < 0.019 and OR = 1.178, 95% CI = 1.033–1.344, *p* = 0.015, respectively].

**Conclusion:**

Early Se supplementation after trauma confers positive results in terms of decreasing overall ICU LOS/hospital LOS and mortality. Organ injury, particularly hepatic insults, and inflammatory status, also recovered better.

## Introduction

Critical diseases, such as trauma, major burn, and sepsis, trigger oxidative stress by increasing the production of reactive oxygen species and reactive nitrogen species, which significantly contributes to systemic inflammatory response syndrome (SIRS), thereby causing vessel/pulmonary endothelial damage, acute respiratory distress syndrome, and multiple organ failure (1, 2). Recent studies have reported that both SIRS and counter-inflammatory response syndrome simultaneously develop in the initial phase following an injury. Depletion of several endogenous micronutrients, such as selenium (Se), ascorbic acid, zinc, glutamine, and tocopherols, is associated with poor prognosis (3–6). These inflammatory processes also impair normal cells and make them evolve toward degradation in numerous clinical conditions, including cardiovascular diseases, cancers, autoimmune disorders, pancreatitis, neurodegenerative disorders, burns, and traumas. Moreover, Kaushal et al. (7) have comprehensively discussed mechanisms focusing on the anti-inflammatory role of Se and their correlations. Several studies have demonstrated that Se plays a key role in regulating immunity and inflammation responses as an essential micronutrient, which is required for more than 25 proteins in the body (8).

Trauma accounts for 1 of the 10 leading causes of death (9). Initial mortality depends on injury severity (10). However, late deaths that developed from days to weeks following an initial injury are associated with inflammatory processes after a traumatic injury (11). Sepsis, acute respiratory distress syndrome, and multiorgan failure are substitutes for the cause of death. Serum concentration of Se and its transport protein “selenoproteins” (mainly as selenocysteine) are extremely low within the first hour of trauma (mean Se: 33.6 μg/L; SELENOP: 1.4 mg/L, trauma patients vs. mean Se: 80.1 μg/L; SELENOP: 4.1 mg/L, healthy general population, Braunstein et al.); a subsequently increased selenoprotein biosynthesis that dominates serum Se concentration occurs at 6–12 h after an injury (mean Se: 48.6 μg/L; SELENOP: 1.9 mg/L) (12). This drastic Se-decline phenomenon may offer an explanation about the benefits of Se supplementation in critical diseases (13) and that supplemental Se may efficiently support selenoprotein biosynthesis during the first hours. A previous study has generally indicated that Se supplementation confers positive effects with less mortality rates and lower infection and multiorgan failure rates (14–16). Recent nutritional therapy guidelines [American Society for Parenteral and Enteral Nutrition (ASPEN)] (17) have suggested that patients should receive trace elements or special nutrients. In in a meta-analysis by Huang et al. (18), they reported a decrease in the length of intensive care unit (ICU) stay and mortality without specific adverse effects. However, ensuring treatment benefits and lung protection were reported as major challenges owing to the small sample size and moderate heterogeneity (*I*2 = 45.496%, *p* < 0.001).

This study aimed to retrospectively review the effectiveness of Se supplementation on mortality, length of ICU/ventilator dependence, and serological changes in major trauma patients.

## Materials and methods

### Study participants and design

**FIGURE 1:**
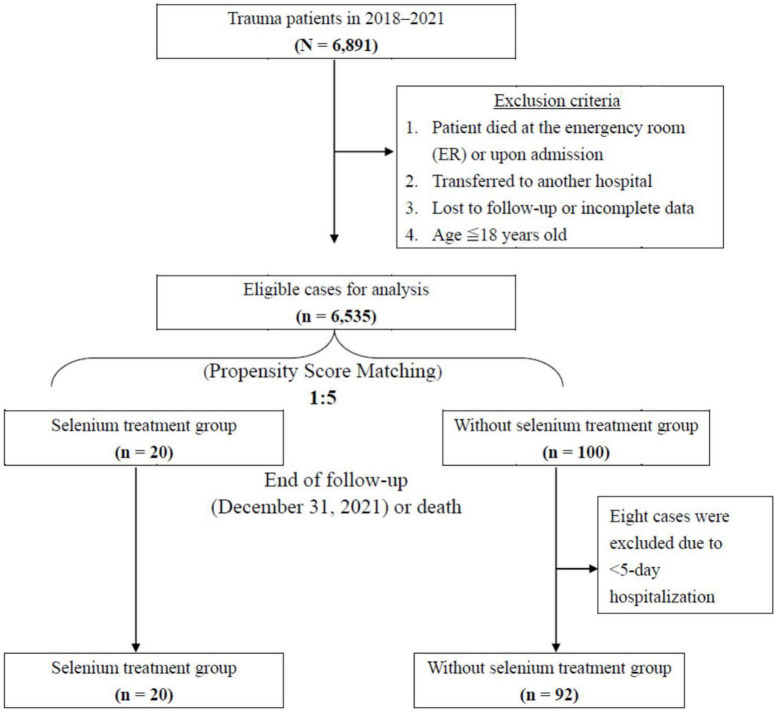
Flowchart of study sample selection from registration in the Tri-Service General Hospital Trauma Data Bank.

A total of 6,891 patients were registered in Tri-Service General Hospital Medical Center Trauma Databank from January 2018 to December 2021. Information on these trauma patients was collected following the selection protocol presented in Figure 1. At the time of resuscitation in the emergency room, written informed consent was obtained from the patients or their closest relatives if they agreed to the Se supplement. Regardless of hemodynamic stability, enteral nutrition establishment, and surgical intervention, intravenous sodium selenite 100 mcg Q8H was administered for 5 consecutive days since the day of ICU admission. The exclusion criteria for the study were as follows: (I) patients who died in the emergency room or upon admission; (II) patients who were transferred to another hospital; (III) patients aged below 18 years; (IV) patients who were lost to follow-up or had incomplete data. A total of 6,535 patients were eligible, and 20 trauma patients who were admitted to the ICU of Tri-Service General Hospital and were receiving Se supplementation were entered in the study group. Patients included in this study were categorized into the following two groups: Se treatment group and non-Se treatment group, based on whether Se supplementation was started in the first consecutive 5 days or not. Subsequently, 1:5 propensity score matching (PSM) analysis was introduced following the exclusion of patients with <5-day hospitalization. Finally, the Se and non-Se treatment groups included 20 and 92 patients, respectively. All covariate imbalances were alleviated. The primary outcome was OS; the secondary outcomes were hospital/ICU length of stay (LOS), serologic change, ventilator dependence days, and ventilation profile.

This retrospective cohort study utilized a single-center trauma databank for data collection. The study was approved by the ethics committee of Tri-Service General Hospital and conducted in accordance with the Declaration of Helsinki.

### Data collection

Data were collected from Tri-Service General Hospital Trauma Data Bank. All data collectors were blinded to the study aims at the time of data abstraction. All medical records were reviewed by three independent physicians. Inconsistent clinical scores were recalculated until consensus was achieved.

Demographic information and nominal variables, including age, sex, alcoholic ingestion, extracorporeal membrane oxygenation (ECMO) use, and comorbidity, were collected. Continuous variable evaluation and clinical scores were also investigated. Trauma and injury severity score (TRISS), revised trauma score (RTS), new injury severity score (NISS), and injury severity score (ISS) (19) were collected to assess the severity of trauma. Glasgow Coma Scale and vital signs, including blood pressure, respiratory rate, and heart rate, were recorded. Moreover, serum albumin, liver function [glutamic oxaloacetic transaminase (GOT) and glutamic pyruvic transaminase (GPT)], neutrophil percentage, C-reactive protein (CRP) level, and Ratio of arterial oxygen partial pressure to fractional inspired oxygen (PaO2/FiO2) ratio at days 1 and 5 were measured for inflammatory response and lung injury severity. ICU LOS, days of weaning of ventilators, and outcomes were surveyed for therapeutic indicators.

### Propensity score matching analysis and statistical analysis

Propensity score matching analysis was performed using the matching package in R software (version 4.0.2 for Windows, Bell Laboratories, Murray Hill, NJ, USA). Logistic regression was used for the estimation algorithm of PSM, and the matching algorithm used was the nearest neighbor matching. The option for nearest neighbor was set as random matching order, non-replacement, no caliper, tolerance was set at 0.15, and 1:5 matching according to age, sex, and ISS. The standardized differences of the matching score were set at 0.20.

Continuous variables were presented as means (±standard deviations). Fisher’s exact test determined the association between categorical variables, as appropriate. The Mann–Whitney *U*-test was used for continuous data, as appropriate. Condition logistic regression analysis of risk factors for mortality and pulmonary complication among propensity-matched trauma patients were also investigated, as appropriate. Statistical analysis was performed using SPSS version 26.0 for Windows (SPSS Inc., Chicago, IL, USA). *P* < 0.05 was considered statistically significant.

## Results

### Baseline characteristics

Baseline characteristics of patients allocated to the Se and without Se treatment groups are described in Table 1. The alcohol exposure percentage (95.0 vs. 71.4%, *p* = 0.019) and thoracic area injury [Abbreviated Injury Scale (AIS): 3.6 ± 0.6 vs. 2.3 ± 1.7, *p* = 0.002] were higher in the Se treatment group than those in the non-Se treatment group. In the non-Se treatment group, the incidence of head and neck area injury (AIS: 3.6 ± 1.3 vs. 2.8 ± 1.0, *p* = 0.003) was higher than that in the Se treatment group; however, no statistical difference in the ISS score was noted (28.8 ± 7.5 vs. 29.3 ± 7.2, *p* = 0.711). Furthermore, no significant differences in age, sex, vital signs on admission, Glasgow Coma Scale, comorbidities, trauma scores (RTS, NISS, TRISS, and ISS), and ECMO support between the two groups were noted. Baseline clinicopathological characteristics and injury severity were generally balanced.

**TABLE 1:** Comparison of variables between patients with or without selenium treatment after 1:5 propensity score matching (PSM).

Variable	Selenium treatment (*n* = 20)	Without selenium treatment (*n* = 92)	*P*-value
Age, y	43.2 ± 18.0	49.8 ± 20.1	0.180
Sex, male	15 (75.0%)	69 (75.0%)	0.623
**Vital signs on admission**			
Systolic blood pressure (mmHg)	123.3 ± 35.5	132.4 ± 29.9	0.461
Respiratory rate	25.9 ± 24.6	20.3 ± 3.4	0.351
Heart rate (bpm)	96.3 ± 25.2	91.1 ± 17.7	0.169
Glasgow Coma Scale	10.6 ± 3.6	10.8 ± 4.2	0.797
With comorbidities	7 (35.0%)	41 (44.6%)	0.467
**Comorbidity category**			
Central nervous system	1 (5.0%)	12 (13.0%)	0.457
Cardiovascular system	4 (20.0%)	24 (26.1%)	0.777
Respiratory system	0 (0%)	3 (3.3%)	0.413
Digestive system	0 (0%)	2 (2.2%)	0.506
Genitourinary system	0 (0%)	1 (1.1%)	0.640
Endocrine system	3 (15.0%)	18 (19.6%)	0.761
Cancer	1 (5.0%)	0 (0%)	0.179
With alcohol exposure	19 (95.0%)	65 (71.4%)	0.019*
RTS	6.67 ± 0.76	6.70 ± 1.33	0.391
NISS	33.7 ± 8.4	32.2 ± 7.9	0.359
TRISS	0.82 ± 0.14	0.76 ± 0.24	0.690
ISS	28.8 ± 7.5	29.3 ± 7.2	0.711
**AIS body region**			
Head and neck	2.8 ± 1.0	3.6 ± 1.3	0.003*
Face	0.2 ± 0.6	0.6 ± 0.9	0.090
Thorax	3.6 ± 0.6	2.3 ± 1.7	0.002*
Abdomen	1.2 ± 1.6	0.8 ± 1.4	0.289
Extremities	1.9 ± 0.9	1.6 ± 1.2	0.383
External	0.3 ± 0.5	0.5 ± 0.7	0.245
Hospital LOS, days	20.0 ± 10.0	37.4 ± 42.0	0.026*
ICU LOS, days	6.8 ± 3.6	13.1 ± 12.6	0.006*
Ventilator dependence, days	6.0 ± 8.4	13.9 ± 17.7	0.259
With ECMO support	1 (5.0%)	1 (1.1%)	0.327
Serum albumin change (%)	–11.55 ± 7.69	–2.89 ± 23.26	0.011*
Neutrophil percentage change (%)	–26.36 ± 9.79	29.65 ± 47.06	< 0.001*
**Liver function change (%)**			
GOT	–62.63 ± 27.53	12.04 ± 161.03	< 0.001*
GPT	–59.38 ± 28.37	67.39 ± 223.45	< 0.001*
CRP change (%)	–71.94 ± 16.57	121.21 ± 264.01	< 0.001*
**Gas exchange**			
PaO2/FiO2 ratio, day 1	277.74 ± 119.17	329.32 ± 161.90	0.012*
PaO2/FiO2 ratio, day 5	488.57 ± 139.73	412.86 ± 152.40	0.053
Outcome, survival	20 (100%)	77 (83.7%)	0.042*

RTS, revised trauma score; NISS, new injury severity score; ISS, injury severity score; AIS, abbreviated injury scale; LOS, length of stay; ICU, intensive care unit; ECMO, extracorporeal membrane oxygenation; GOT, glutamic oxaloacetic transaminase; GPT, glutamic pyruvic transaminase; CRP, C-reactive protein; PaO2/FiO2, Ratio of arterial oxygen partial pressure to fractional inspired oxygen.

*Significant difference.

### Comparison of clinical outcomes between the two groups

**FIGURE 2:**
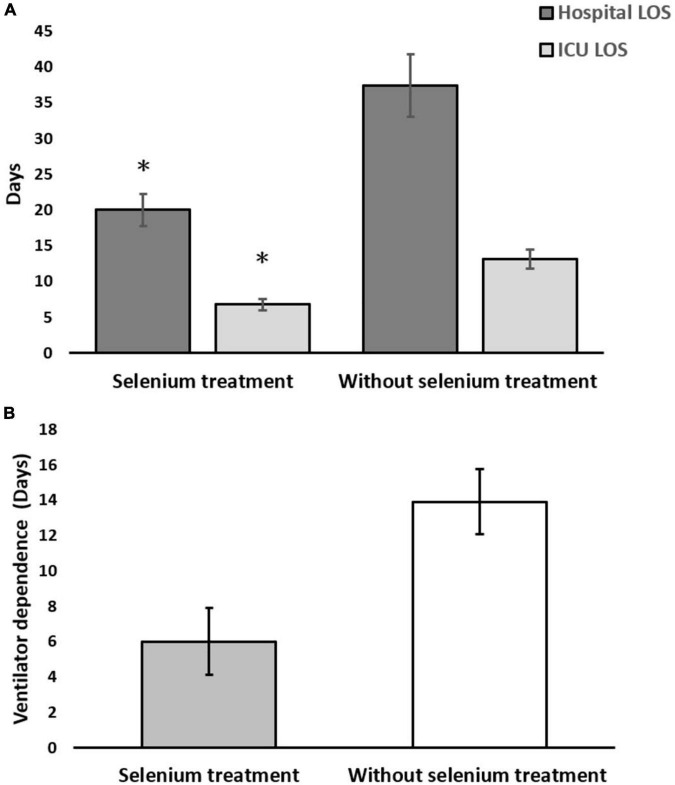
**(A)** Hospital and intensive care unit (ICU) length of stay (LOS); **(B)** ventilator-dependent days in the selenium treatment and non-selenium treatment group. *p < 0.05.

**FIGURE 3:**
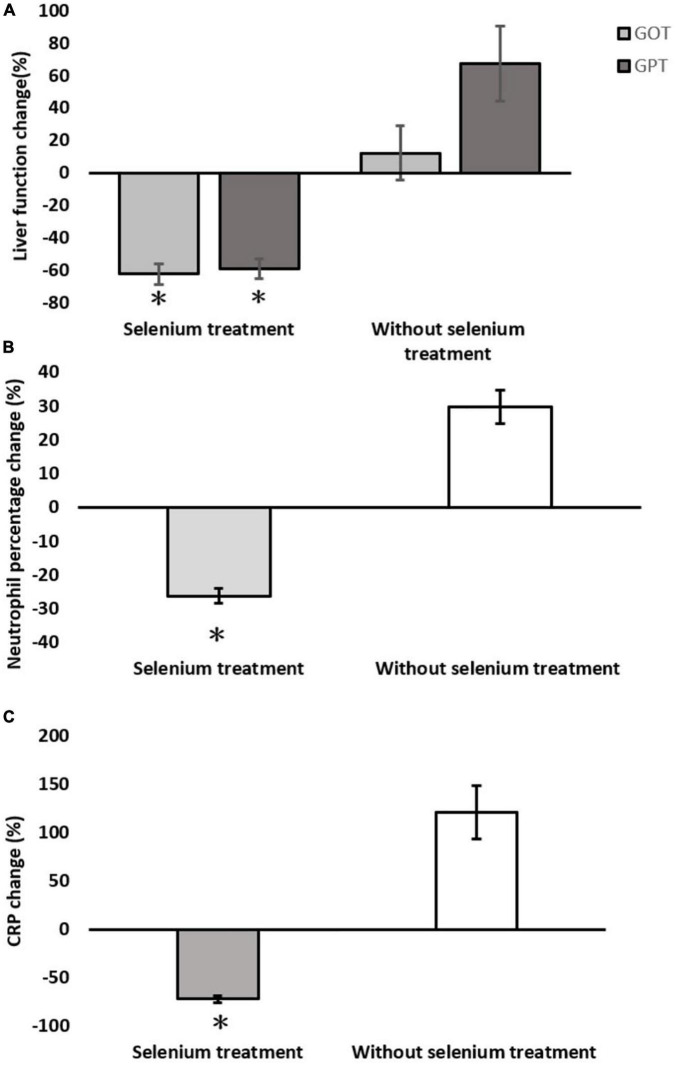
Serological changes between the selenium treatment and non-selenium group. **(A)** Liver function change; **(B)** neutrophil percentage change; **(C)** C-reactive protein (CRP) change. *p < 0.05.

In the matched cohort, the clinical outcomes of the two groups are shown in the lower part of Table 1. The hospital LOS (20.0 ± 10.0 vs. 37.4 ± 42.0 days, *p* = 0.026) and ICU LOS (6.8 ± 3.6 vs. 13.1 ± 12.6 days, *p* < 0.006) revealed significantly fewer number of days in the Se treatment group than those in the non-Se treatment group (Figure 2A). Although ventilator dependence days were less in the Se treatment group (6.0 ± 8.4 vs. 13.9 ± 17.7 days), this did not reach statistical significance (*p* = 0.259) (Figure 2B). Regarding serology, improvement of neutrophil left shift percentage (−26.36 ± 9.79 vs. 29.65 ± 47.06%, *p* < 0.001), liver function (GOT: −62.63 ± 27.53 vs. 12.04 ± 161.03%, *p* < 0.001; GPT: −59.38 ± 28.37 vs. 67.39 ± 223.45%, *p* < 0.001), and CRP level change percentile (−71.94 ± 16.57 vs. 121.21 ± 264.01%, *p* < 0.001) all indicated better outcomes in the Se treatment group (Figures 3A–C); however, serum albumin decline was more prominent in the Se treatment group (−11.55 ± 7.69 vs. −2.89 ± 23.26%, *p* = 0.011). The proportion of gas exchange was lower on day 1 admission (277.74 ± 119.17 vs. 329.32 ± 161.90, *p* < 0.012); however, it became better on day 5 of hospitalization in the Se treatment group (488.57 ± 139.73 vs. 412.86 ± 152.40, *p* = 0.053). In summary, the overall survival (OS) rate in the Se treatment group showed superior results than that in the Se treatment group (100 vs. 83.7%, *p* = 0.042).

### Risk stratifications of mortality and pulmonary complications

We thereafter performed univariate and multivariate logistic regression analyses for risk stratifications of mortality and pulmonary complications (Tables 2, 3). Regression analysis revealed a value of 0.000 in the Se treatment group because there were no mortality cases. Multivariable logistic regression analysis revealed that longer ventilator dependence was positively correlated with higher risk of mortality and pulmonary complications [odds ratio (OR) = 1.262, 95% confidence interval (CI) = 1.039–1.532, *P* < 0.019 and OR = 1.178, 95% CI = 1.033–1.344, *P* = 0.015, respectively]. Furthermore, hospital LOSs were negatively associated with pulmonary complications and mortality in both univariate and multivariate analyses (OR for mortality: 0.712, 95% CI = 0.551–0.920, *P* < 0.010; OR for pulmonary complications: 0.793, 95% CI = 0.873–0.933, *P* < 0.005). Overall, no correlations between demographic factors and either pulmonary complications or mortality, including age, sex, comorbidity, ventilation, ISS score, and ICU LOS, were noted.

**TABLE 2:** Demographic and risk factors for mortality in trauma patients.

Variable	Crude OR (95% CI)	*P*-value	Multivariable OR (95% CI)	*P*-value
Group (Selenium treatment)	0.000		0.000	
Age	1.027 (0.996–1.058)	0.084	1.063 (0.986–1.145)	0.111
Sex, male	5.400 (0.677–43.085)	0.111	0.075 (0.001–3.800)	0.196
**Comorbidity**				
Central nervous system	2.175 (0.523–9.038)	0.285	13.628 (0.564–329.497)	0.108
Cardiovascular system	1.609 (0.499–5.189)	0.426	9.938 (0.304–325.065)	0.197
Respiratory system	3.393 (0.288–39.918)	0.331	17.906 (0.014–39.192)	0.854
Digestive system	6.857 (0.405–115.960)	0.182	5.249 (0.701–188.452)	0.971
Genitourinary system	No mortality	0.999	No mortality	0.999
Endocrine system	0.632 (0.131–3.038)	0.566	0.026 (0.001–4.911)	0.172
Cancer	No mortality	0.999	No mortality	0.999
PaO2/FiO2 ratio, day 1	0.998 (0.995–1.002)	0.366	0.997 (0.982–1.013)	0.719
PaO2/FiO2 ratio, day 5	0.998 (0.994–1.001)	0.210	1.010 (0.992–1.027)	0.275
ISS	1.065 (0.993–1.143)	0.077	1.084 (0.909–1.294)	0.370
ICU LOS, days	0.996 (0.947–1.047)	0.876	1.238 (0.878–1.746)	0.224
Hospital LOS, days	0.947 (0.902–0.994)	0.026*	0.712 (0.551–0.920)	0.010*
Ventilator dependence, days	1.007 (0.977–1.038)	0.641	1.262 (1.039–1.532)	0.019*

OR, odds ratio; CI, confidence interval; RTS, revised trauma score; ISS, injury severity score; LOS, length of stay; ICU, intensive care unit; PaO2/FiO2, Ratio of arterial oxygen partial pressure to fractional inspired oxygen.

*Significant difference.

**TABLE 3:** Demographic risk of pulmonary complications in trauma patients.

Variables	Crude OR (95% CI)	*P*-value	Multivariable OR (95% CI)	*P*-value
Group (Selenium treatment)	0.000		0.000	
Age	1.027 (0.996–1.058)	0.084	1.036 (0.986–1.088)	0.157
Sex, male	5.400 (0.677–43.085)	0.111	0.200 (0.016–2.435)	0.207
Respiratory comorbidity	3.393 (0.288–39.918)	0.331	11.979 (0.038–425.032)	0.703
PaO2/FiO2 ratio, day 1	0.998 (0.995–1.002)	0.366	1.000 (0.988–1.011)	0.973
PaO2/FiO2 ratio, day 5	0.998 (0.994–1.001)	0.210	1.003 (0.992–1.027)	0.763
ISS	1.065 (0.993–1.143)	0.077	1.026 (0.913–1.153)	0.667
ICU LOS, days	0.996 (0.947–1.047)	0.876	1.121 (0.939–1.338)	0.208
Hospital LOS, days	0.947 (0.902–0.994)	0.026*	0.793 (0.873–0.933)	0.005*
Ventilator dependence, days	1.007 (0.977–1.038)	0.641	1.178 (1.033–1.344)	0.015*

OR, odds ratio; CI, confidence interval; RTS, revised trauma score; ISS, injury severity score; LOS, length of stay; ICU, intensive care unit; PaO2/FiO2, Ratio of arterial oxygen partial pressure to fractional inspired oxygen.

*Significant difference.

## Discussion

This retrospective study disclosed that trauma patients can benefit from early phase utilization of Se supplement. Mortality rate, ICU LOS, hospital LOS, systemic inflammation (CRP and neutrophil downgrade), and acute hepatitis were significantly reduced. Although data on ventilator dependence and post-treatment ventilation (day 5 gas exchange) did not achieve statistical significance, they displayed pulmonary protective tendency in the Se supplement group.

Consistent with our results, the results of several previous studies have also presented positive correlations between low initial Se levels and adverse outcomes in critically ill patients (20–23). Moreover, in a recently published meta-analysis by Jen-Fu Huang (24), it has been reported that Se supplementation after trauma confers positive effects in decreasing mortality and ICU and hospital LOSs. Six randomized controlled trials or retrospective studies were included in this systematic review, and doses of Se were 200–500 mcg per day. It appeared safe, and major side effects were not observed.

Compared with other systemic inflammatory diseases, a study in Berlin in 2019 (12) revealed dramatic changes in serum Se and selenoprotein concentrations in trauma patients; however, this was in contrast to the low Se status observed in patients with sepsis, wherein a slow and gradual decline in serum biomarkers was observed. Therefore, this may offer an explanation for the observed beneficial effects of supplemental Se in critical illness (13), where some additional Se may efficiently help selenoprotein biosynthesis during the first hours after the traumatic injury.

Substantial Se-based micronutrient supplementation is currently being widely used for patients with critical illness, cancer, or neurodegenerative disorders. However, various cytotoxicity and adverse effects have been reported. A study by Wenyi Zheng (25) proposed extracellular albumin binding as a predominant factor in determining the cytotoxicity of selenocompounds (SeCs). The available evidence of SeC cytotoxicity is limited to intracellular factors. In comparison, scattered information suggests that SeCs show strong albumin binding; therefore, cellular uptake and downstream cytotoxicity, which are orchestrated by extracellular albumin, are hypothesized. Based on this study, we could explain that a remarkable decrease in albumin levels in the Se supplementation group may contribute to extracellular albumin binding for additional SeCs on their cellular uptake and cytotoxicity.

Selenium deficiency may cause reduced glutathione peroxidase activity that indirectly regulates the expression of cyclooxygenases and lipoxygenases *via* the mitogen-activated protein kinase pathway and cyclooxygenases-2, by controlling the nuclear factor kappa-light-chain-enhancer of activated B-cells. These enzymes are involved in the production of lipid mediators, such as prostaglandins, thromboxanes, prostacyclins, leukotrienes, and oxidized fatty acids, which are well-known inflammatory biomarkers from tissues and immune cells in response to stress, free radicals, and infections. Moreover, these molecules are key factors in the modulation of pivotal metabolic signaling pathways and convert pro-inflammatory macrophages (M1) to anti-inflammatory macrophages (M2). Increasing Se concentrations in patients with critical illness, for example, is reported to be associated with the decrease in pro-inflammatory cytokines [interleukin (IL)-6, IL-8, and IL-17] and the increase in the Se concentration in protein metabolites, which are expressed as antioxidant selenoproteins [e.g., glutathione peroxidases and selenoprotein thioredoxin reductase (TXNRD1)]. Selenium is essential for selenoenzyme biosynthesis and functional activity. Previously identified TXNRD1, which is mainly expressed by airway epithelia, is a promising therapeutic target for pulmonary injury prevention, most likely *via* nuclear factor E2-related factor 2 (Nrf2)-dependent mechanisms. A recent study (25) showed that Se levels positively correlated with Nrf2 and selenoprotein activation following TXNRD1 inhibition. These data indicate that Se levels significantly influence physiologic responses to TXNRD1 inhibitors. In summary, clinical Se deficiency must be corrected for the optimal therapeutic effectiveness of TXNRD1 inhibitors in the prevention of lung disease.

A guideline published by the ASPEN in collaboration with the Society of Critical Care Medicine concludes that a recommendation regarding Se supplementation in sepsis cannot be made because of conflicting studies (26). In contrast, the Cochrane Collaboration (27) and Landucci et al. (28) both conducted systematic reviews of Se supplementation in ICU patients and concluded that parenteral supplementation with high-dose Se would be effective in reducing mortality in critically ill patients. However, these reviews were conducted using different designs, and heterogenicity is high. Thus, high-quality prospective randomized control trials to evaluate traumatic critical illness patients are warranted.

This retrospective review revealed that early Se supplementation after trauma confers positive results in decreasing the overall lengths of ICU and hospital stays and mortality. In addition, it seemed to have lung-protective tendencies. Organ injury, particularly hepatic insults and inflammatory status, also recovered better than the control group under balanced severity of illness, comorbidity, and baseline characteristics. Additional randomized control studies and subgroup studies to elucidate the concentration effect are needed, especially on the pulmonary protection effect.

### Limitations

This study has some limitations. The major limitation of this study is its retrospective nature, which is associated with information and selection bias. To achieve an ideal random effect, we selected patients as study groups based on their willingness to take Se supplements rather than physician consideration. Selection bias from unknown confounders was inevitable given the difficulty of the trauma scenario. In addition, the serum level of Se was not measured before, during, or after treatment. In Taiwan, the average serum Se concentrations of males and females are 112.9 ± 20.5 and 109.4 ± 19.8 μg/L, respectively (29), indicating optimal serum levels (>100 μg/L). In contrast, we have observed a strong deficit of Se and selenoproteins (12) at the early stage of trauma. We used 1:5 PSM analysis to minimize bias in the abovementioned factors, including baseline demographics, intervention, medication, and nutrition status. The criteria for Se supplementation could be made for a specific type or group of injury for subgroup and secondary analysis. Additionally, consecutive serum concentrations could be collected for data and clinical implications correlation. However, the results enhance the generalizability of this study as it reflects the current real-world practice. The large sample size generated from this single-center study also allowed robust statistical analysis, which is important when we are studying a particular treatment where the expected difference is uncertain. To more precisely determine the role of early Se supplementation in trauma patients, a large prospective study is needed.

## Data availability statement

The original contributions presented in this study are included in the article/supplementary material, further inquiries can be directed to the corresponding author.

## Ethics statement

The studies involving human participants were reviewed and approved by Ethics Committee of Tri-Service General Hospital. Written informed consent for participation was not required for this study in accordance with the national legislation and the institutional requirements.

## Author contributions

Y-CC, C-ML, Z-JH, and S-DH conceptualized and wrote the manuscript. C-HC and W-CC were responsible for data curation and formal analysis. All authors read and agreed to the published version of the manuscript.
